# Enhanced OER Performances of Au@NiCo_2_S_4_ Core-Shell Heterostructure

**DOI:** 10.3390/nano10040611

**Published:** 2020-03-27

**Authors:** Yuepeng Lv, Sibin Duan, Yuchen Zhu, Peng Yin, Rongming Wang

**Affiliations:** Beijing Advanced Innovation Center for Materials Genome Engineering, Beijing Key Laboratory for Magneto-Photoelectrical Composite and Interface Science, School of Mathematics and Physics, University of Science and Technology Beijing, Beijing 100083, China; b20150331@xs.ustb.edu.cn (Y.L.); sibinduan@ustb.edu.cn (S.D.); cy931122@live.com (Y.Z.); yinpeng0537@163.com (P.Y.)

**Keywords:** metal-semiconductor, core-shell, bimetallic sulfide, OER, structure-property relationship

## Abstract

Transition metal sulfides have attracted a lot of attention as potential oxygen evolution reaction (OER) catalysts. Bimetallic sulfide possesses superior physicochemical properties due to the synergistic effect between bimetallic cations. By introducing a metal-semiconductor interface, the physicochemical properties of transition metal sulfide can be further improved. Using the solvothermal method, Au@NiCo_2_S_4_ core-shell heterostructure nanoparticles (NPs) and bare NiCo_2_S_4_ NPs were prepared. The measurement of the OER catalytic performance showed that the catalytic activity of Au@NiCo_2_S_4_ core-shell heterostructure was enhanced compared to bare NiCo_2_S_4_ NPs. At the current density of 10 mA cm^−2^, the overpotential of Au@NiCo_2_S_4_ (299 mV) is lower than that of bare NiCo_2_S_4_ (312 mV). The Tafel slope of Au@NiCo_2_S_4_ (44.5 mV dec^−1^) was reduced compared to that of bare NiCo_2_S_4_ (49.1 mV dec^−1^), indicating its faster reaction kinetics. Detailed analysis of its electronic structure, chemical state, and electrochemical impedance indicates that the enhanced OER catalytic performances of bare Au@NiCo_2_S_4_ core-shell NPs were a result of its increased proportion of high-valance Ni/Co cations, and its increased electronic conductivity. This work provides a feasible method to improve OER catalytic performance by constructing a metal-semiconductor core-shell heterostructure.

## 1. Introduction

Due to the limited supply and the threat to the environment of fossil fuels, renewable and clean energies need to be developed. Hydrogen is an ideal energy substitute, with high energy density and zero carbon emission. Electrochemical water splitting is an environmentally friendly and promising method for hydrogen production [1,2]. However, the working potential of electrochemical water splitting is much higher than 1.23 V—the theoretical potential [3,4]. Reducing the overpotential and enhancing the stability of catalytic materials are the main research goals of electrochemical water splitting. OER as the anode of the electrochemical water splitting reaction is a multi-step reaction that requires high energy to form O–O* bonds, leading to high overpotential and severely limiting hydrogen production efficiency [5]. Therefore, efficient catalysts are necessary to reduce the overpotential and enhance the OER reaction rate. IrO_2_ and RuO_2_ are recognized as the most effective OER catalysts, but the low supply and high price of these catalysts seriously limit their commercial applications [6,7,8].

Transition metals—with their nitrides, phosphides, oxides, hydroxides, sulfides, and selenides—are widely recognized as effective OER catalysts [4,9,10,11,12,13,14]. Transition metal sulfide is widely used in the field of OER catalysis due to its abundant supply, low price, superior catalytic activity, and stable performance. Co/Ni sulfides have been considered as potential catalysts for their multivalent properties and tunable electronical structure [15]. Compared with monometallic sulfides, bimetallic sulfides exhibit outstanding OER activities because they have more active sites, higher conductivity, larger electrochemical surface area, and higher structural stability [13,16,17,18,19,20,21,22,23,24,25,26,27]. Among the bimetallic catalysts, bimetallic nickel cobalt sulfides have become one of the most studied bimetallic structure compounds, due to their advantages of high conductivity and the redox reaction in which Ni and Co elements are more abundant in multi-valence than monometallic sulfides [22,28,29]. For example, (Ni, Co)S_2_ nanosheet has a lower overpotential of 270 mV, compared to CoS_2_ nanosheet (350 mV), and NiS_2_ nanosheet (410 mV) at 10 mA cm^−2^, which is due to its good mass/electron transfer properties, more catalytic active sites and facilitated adsorption to O* [30].

To further enhance the OER catalytic properties and promote the application of bimetallic sulfides, designing heterostructures composed of bimetallic sulfide and metal is one of the most effective methods [31,32,33,34,35]. The enhanced physicochemical properties of metal-semiconductor heterostructures are mainly due to the interaction between metal and semiconductor [36,37]. Compared with yolk-shell and oligomer-like heterostructures, the core-shell heterostructure has the maximum contact interface, which can effectively enhance electron transmission along the interface, improve the electrical conductivity, and optimize the electronic structure [9,37,38]. For example, Au@NiS*_x_* core-shell heterostructure has higher HER activity than Au-NiS*_x_* yolk-shell and Au-NiS*_x_* oligomer-like NPs. This is because the Au@NiS*_x_* core-shell heterostructure has a larger contact interface, leading to promoted charge transfer and higher conductivity [39]. The OER activity of Au/Ni_12_P_5_ core-shell heterostructure has also been demonstrated to be improved by the electron interaction between Au and Ni_12_P_5_ [36].

Herein, Au@NiCo_2_S_4_ heterostructure and bare NiCo_2_S_4_ NPs were rationally designed and prepared through solvothermal methods. Using time-dependent experiments, the evolution mechanism of Au@NiCo_2_S_4_ NPs was studied. The measurements of the OER performance indicate that Au@NiCo_2_S_4_ core-shell heterostructure shows a high current density of 10 mA cm^−2^, at a lower overpotential of 299 mV, and a lower Tafel slope of 44.5 mV dec^−1^, compared with bare CoNi_2_S_4_ NPs.

## 2. Materials and Methods 

### 2.1. Preparation of the Au@NiCo_2_S_4_ Core-Shell NPs

**Figure 1:**
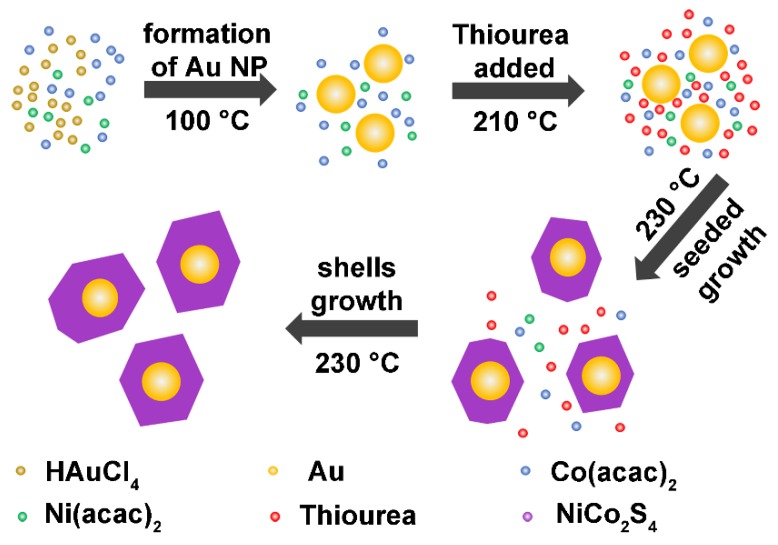
Diagram for the synthesis processes of Au@NiCo_2_S_4_ NPs. The core-shell structure was formed by the seeded growth of NiCo_2_S_4_ shells on Au NPs.

Au@NiCo_2_S_4_ core-shell heterostructure NPs were controllably synthesized using the seeded growth method. The synthesis method is shown in Figure 1. Typically, 0.05 g Ni(acac)_2_ and 0.1 g Co(acac)_2_ were dissolved in 16 mL oleylamine (OAm) in a three-necked flask. The solution was heated to 100 °C to form a clear solution. An amount of 3 ml toluene solution containing 0.03 g HAuCl_4_∙4H_2_O was dropped into the solution afterwards. Au NPs were formed at 100 °C in the OAm by the reduction of the Au precursor, which were used as seeds during the subsequent growth of NiCo_2_S_4_ shells. The solution was kept at this temperature for 1 h and then heated to 210 °C. 0.123 g thiourea was added to the solution at 210 °C to serve as a sulfur source. The solution was then heated to 230 °C, and held at this temperature for 3 h. During this time, the NiCo_2_S_4_ shell formed and grew on the surface of the Au seeds. Finally, the solution was cooled to room temperature. The prepared sample was washed with a mixture of chloroform and acetone and collected by centrifugation. The solution was stirred during the whole reaction, and Ar gas was injected as the protective gas.

### 2.2. Preparation of the Bare NiCo_2_S_4_ NPs

The bare NiCo_2_S_4_ NPs were prepared by the typical solvothermal method. Amounts of 0.05 g Ni(acac)_2_ and 0.1 g Co(acac)_2_ were dissolved in 16 mL OAm in a three-necked flask. The solution was heated to 210 °C to form a clear solution. 0.123 g thiourea was added to the solution afterwards. The solution was then heated to 230 °C, and kept at this temperature for 3 h. After the solution was cooled to room temperature, the prepared sample was washed with a mixture of chloroform and acetone, and collected by centrifugation. The solution was stirred during the whole process, and Ar gas was injected as a protective gas.

### 2.3. Material Characterization

The crystal structures of the products were characterized by X-ray diffraction (XRD) using a Rigaku D/max 2200 PC diffractometer, with Cu Kα radiation (λ = 0.15406 nm). An Oxford X-Max80T energy dispersive X-ray spectrograph was used to measure the elemental composition and distribution of the products. A JEOL JEM-2200FS field emission transmission electron microscope (TEM, Tokyo, Japan, JEOL) was used to investigate the morphology of the samples. The atomic arrangements and microstructure of the as-grown samples were examined on a Titan ETEM G2 aberration-corrected transmission electron microscope (AC-TEM). A ULVAC-PHI Phi5000 VersaProbe III X-ray photoelectron spectrograph (XPS, Chigasaki, Japan, ULVAC-PHI) was used to measure the electronic structure and valence of the elements. 

### 2.4. OER Catalytic Measurements

A CHI 760E electrochemical workstation was used to measure the OER catalytic performance of the as-prepared samples. The measurements were performed in a 1 M KOH solution, using a three-electrodes system. To prepare the working electrodes, a 5 mg sample was dispersed into 1 mL ethanol and stirred magnetically for 20 min. Then, 5 mg carbon black was added to the solution and stirred for 20 min. 100 μL 5 wt % Nafion was added to the solution, and the homogeneous ink was formed by stirred magnetically. 44 μL ink was dropped onto the surface of a piece of carbon paper (1 cm^2^) and dried at room temperature to serve as the working electrode. About 0.2 mg of the sample was loaded onto the working electrode. The Hg/HgO and Pt plate were used as the reference electrode and counter electrode, respectively. Linear sweep voltammetry (LSV) was used to characterize OER catalytic performance, with a scan rate of 2 mV s^−1^, and 95% iR compensation. The electrochemical impedance spectroscopy (EIS) of the samples was carried out by AC impedance, with a frequency range from 100,000 to 0.1 Hz. The stability of the samples was characterized by a current-time curve.

## 3. Results and Discussion

Core-shell NPs with a single crystal structure are difficult to be prepare because of the big differences in crystal structures and physicochemical properties between metal and sulfide [35]. Understanding the growth mechanism is helpful for their preparation and controlling their electrocatalytic performance. The time-dependent experiments were conducted in combination with with TEM and EDS analyses. Samples were collected from 0 to 180 min after thiourea was added. Figure S1a–f shows the morphologies of the collected samples. During the formation of Au@NiCo_2_S_4_ core-shell NPs, the Au NPs kept a five-fold symmetric morphology, and the size of Au NPs was also maintained at 8~10 nm. The shells gradually formed and thickened to about 10 nm. Table S1 shows the atomic ratios of Au to Co gradually decrease from 1 to 0.21. From 10 to 30 min, the atomic ratios of Co to S changed from 0.49 to 0.42. From 30 to 180 min, the atomic ratios of Co to S barely changed. This indicates that the sulfuration process has been completed at 30 min. The above process indicates that the evolution mechanism of Au@NiCo_2_S_4_ NPs is a typical seeded growth of NiCo_2_S_4_ shells on the Au NPs.

**Figure 2:**
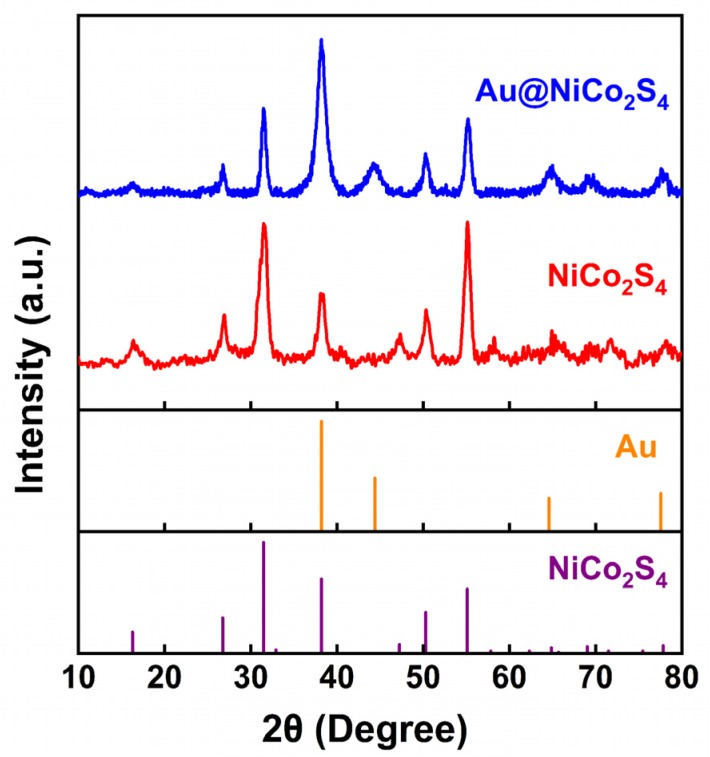
XRD patterns of as-prepared Au@NiCo_2_S_4_, and bare NiCo_2_S_4_ NPs.

Figure 2 shows the XRD spectra of Au@NiCo_2_S_4_ and bare NiCo_2_S_4_ NPs, which confirms that the samples consist of cubic NiCo_2_S_4_ (JCPDS No. 43-1477) and cubic Au (JCPDS No. 99-0056). The impurity phase was not detected in the XRD patterns, indicating the high purity of the as-grown sample. Scherrer’s equation (*d = 0.89λ/(β·cosθ_B_)*, where *λ* is the X-ray wavelength, *θ_B_* is the Bragg diffraction angle and *β* is the full width at half maxima of the peak) was used to estimate the crystal sizes of the products. The crystal size of NiCo_2_S_4_ was calculated to be about 11.8 nm, using the peak of NiCo_2_S_4_ (311) at about 31.5°, and that of the peak of Au was calculated to be about 8.8 nm, using the peak of Au (111) at about 38.2°.

The chemical composition of the synthesized samples was confirmed by the EDS measurements. The EDS spectra of Au@NiCo_2_S_4_ and bare NiCo_2_S_4_ NPs show that the products were composed of S, Co, Ni, and Au, as shown in Figure S2. The atomic ratio for S, Co, Ni, and Au for the Au@NiCo_2_S_4_ and bare NiCo_2_S_4_ NPs is also illustrated in Table S2. The TEM specimen holder may cause the additional signals of Si and Cu. The atomic percentages of Ni, Co, and S of the Au@NiCo_2_S_4_ heterostructure are 10.1%, 24.7%, and 59.9%, respectively. The atomic percentages of Ni, Co, and S of the bare NiCo_2_S_4_ NPs are 11.4%, 25.5%, and 63.1%, respectively. Compared with the stoichiometric proportion, the contents of S are slightly higher, possibly due to the adsorption of additional S on the surface of the NPs.

**Figure 3:**
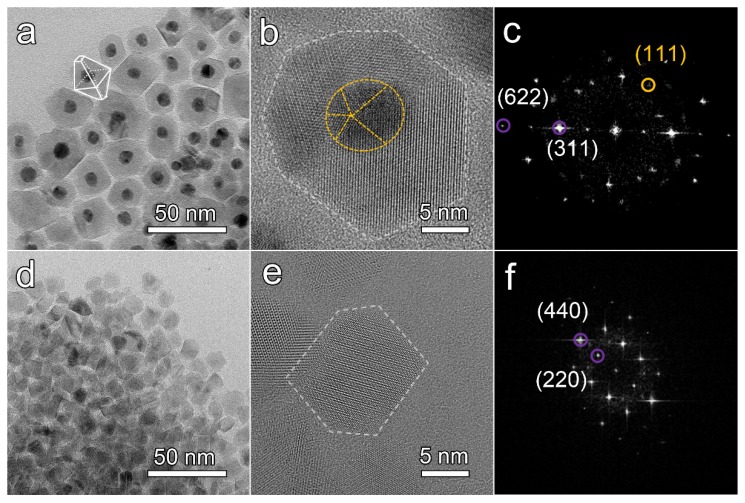
(**a**) Typical bright field TEM images of Au@NiCo_2_S_4_ NPs, (**b**) HRTEM images obtained by AC-TEM of an Au@NiCo_2_S_4_ NP, (**c**) FFT pattern of a Au@NiCo_2_S_4_ NP, (**d**) Typical bright field TEM images of NiCo_2_S_4_ NPs, (**e**) HRTEM images obtained by AC-TEM of a bare NiCo_2_S_4_ NP, (**f**) FFT pattern of a bare NiCo_2_S_4_ NP. In the FFT pattern, the diffraction spots of Au and NiCo_2_S_4_ are marked by yellow and purple circles, respectively.

The morphologies and microstructures of the Au@NiCo_2_S_4_ and the bare NiCo_2_S_4_ NPs were characterized by TEM. As shown in Figure 3a, the interface between the darker Au cores and the lighter NiCo_2_S_4_ of the NPs is clean and smooth, without impurity phases, pores, layers or amorphous structure. The bonding between the Au cores and NiCo_2_S_4_ shells was good along the interface because no additional diffraction contrast from the strain was detected. The diameter of the cores is 8.2 ± 1.5 nm. The shell with lighter contrast has a polyhedral structure, with dimensions of 21.9 ± 5.5 nm. The morphologies of these NPs are mainly truncated octahedrons. In Figure S3a–e, the core-shell heterostructure was further confirmed by rotating the TEM samples. Regardless of the viewing angle, the Au NPs were always located at the center of the particles, indicating that the prepared sample was core-shell structure rather than Au NPs supported on the surface of NiCo_2_S_4_ NPs [39]. The atomic arrangements in the Au@NiCo_2_S_4_ NPs were investigated using AC-TEM. An Au@NiCo_2_S_4_ NP with a typical truncated octahedron morphology is shown in Figure 3b. At the atomic scale, no amorphous layer or impurity phase is observed, and the crystal lattice fringe of the NiCo_2_S_4_ shell layer is clear, indicating that Au@NiCo_2_S_4_ NPs crystallize well. The diameter of the Au core is ~ 9 nm, and the thickness of the NiCo_2_S_4_ shell is ~ 9 nm. The FFT pattern (Figure 3c) indicates that the shell of the NP is a cubic phase NiCo_2_S_4_ single crystal. The lattice spacing is 0.285 nm, corresponding to the NiCo_2_S_4_ (311) planes. To confirm the element distribution of Au@NiCo_2_S_4_ NPs, EDS mapping spectra were collected. The spectra shown in Figure S4 indicate that the Au element is only distributed in the core, while the other three elements are distributed in the shell. The polyhedron NiCo_2_S_4_ NPs are exhibited in Figure 3d for comparison. The size of bare NiCo_2_S_4_ NPs is 10.8 ± 2.1 nm. Figure 3e shows a typical NiCo_2_S_4_ polyhedron NP with almost perfect facets. The crystal lattice of the bare NiCo_2_S_4_ NP is clear. The FFT analysis (Figure 3f) demonstrates that the bare NiCo_2_S_4_ NP is a cubic phase NiCo_2_S_4_ single crystal. The lattice spacing was measured to be 0.331 nm, corresponding to the NiCo_2_S_4_ (220) plane. All the above results confirm that Au@NiCo_2_S_4_ NPs is core-shell structured, with single crystal shells.

**Figure 4:**
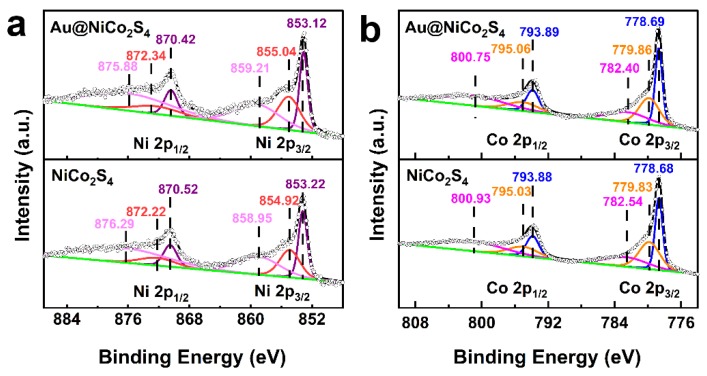
High resolution XPS spectra for the (**a**) Ni 2p, (**b**) Co 2p of Au@NiCo_2_S_4_, and NiCo_2_S_4_ NPs.

The XPS spectra were used to characterize the chemical states and electronic structures of Au@NiCo_2_S_4_ and bare NiCo_2_S_4_ NPs. The full survey scan XPS spectra are shown in Figure S5. By comparing the spectra, it can be confirmed that the full survey scan XPS spectrum of Au@NiCo_2_S_4_ is similar to that of bare NiCo_2_S_4_, except that the peak of Au 4f appears at 80–90 eV. Figure 4 and Figure S6 show high-resolution spectra of the Ni, Co, S, and Au of Au@NiCo_2_S_4_ and bare NiCo_2_S_4_ NPs, respectively. The Ni 2p high-resolution spectra are shown in Figure 4a. The fitting peaks positioned at 853.12–853.22 eV and 870.42–870.52 eV are assigned to Ni^2+^, while the peaks at 854.92–855.04 eV and 872.22–872.34 eV correspond to Ni^3+^ [19]. The Co 2p high-resolution spectra are shown in Figure 4b. The fitting peaks positioned at 778.68–778.69 eV and 793.88–793.89 eV are assigned to Co^3+^, while the peaks at 779.83–779.86 eV and 795.03–795.06 eV correspond to Co^2+^ [19]. Figure S5a shows the S 2p XPS spectra. The peaks positioned at 161.54–161.58 eV and 162.46–162.53 eV are assigned to S^2−^, while the peaks at 162.94–163.00 eV and 163.84–163.97 eV are assigned to S^−^. The peaks at 165.95–166.29 eV are assigned to SO_4_^2−^/SO_3_^2−^, which may be caused by oxidation of the surface sulfur [40]. The Au 4f XPS spectrum of the Au@NiCo_2_S_4_ is shown in Figure S5b.; the fitting peaks positioned at 84.16 eV and 87.81 eV are assigned to metallic Au, and the peaks at 85.19 eV and 88.74 eV can be assigned to Au^+^ [41]. These results indicate that Ni and Co atoms possess multi-valence in the bimetallic sulfides. The ratios of valence states are shown in Table S3. Compared with bare NiCo_2_S_4_, the ratio of Ni^3+^:Ni^2+^ in Au@NiCo_2_S_4_ was increased, which is similar to the trend of Co^3+^:Co^2+^. These results indicate that there is strong electron interaction between Au and NiCo_2_S_4_. Due to the addition of Au NPs, the proportion of Co^3+^ and Ni^3+^ in NiCo_2_S_4_ NPs increases, which is beneficial to their electrochemical performances, because Ni^3+^/Co^3+^ have been considered as active sites during the oxygen evolution [6].

**Figure 5:**
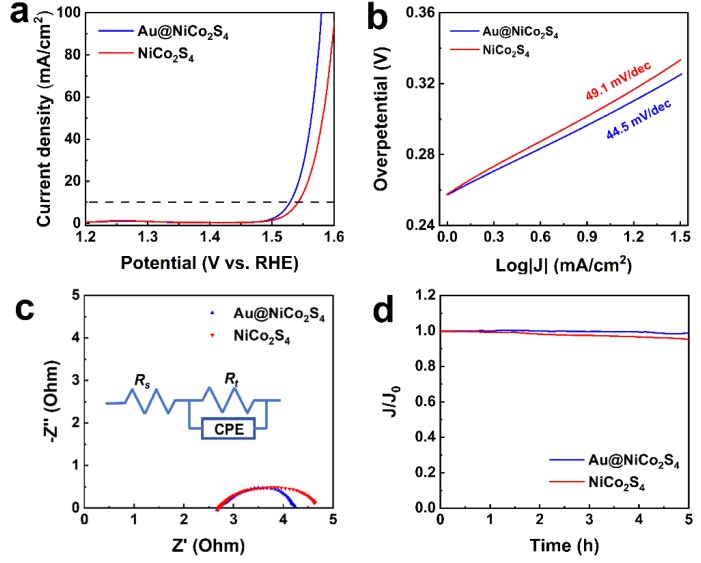
OER performances of Au@NiCo_2_S_4_, and bare NiCo_2_S_4_ NPs in 1 M KOH: (**a**) polarization curves (**b**) Tafel curves, (**c**) Nyquist plots, and (**d**) time–current curves.

The OER catalytic performances of the Au@NiCo_2_S_4_ heterostructure and bare NiCo_2_S_4_ NPs were tested in alkaline solutions. The OER catalytic parameters are shown in Table S4. Figure 5a shows the polarization curves of the as-prepared samples. The overpotential of Au@NiCo_2_S_4_ NPs (299 mV) is lower than that of bare NiCo_2_S_4_ NPs (312 mV) at a current density of 10 mA cm^−2^. The OER catalytic performance is improved by the interaction between Au NPs and NiCo_2_S_4_ shells. The overpotential of Au@NiCo_2_S_4_ NPs is comparable to the value of previously reported catalysts such as NiCo_2_S_4_@g-C_3_N_4_–CNT (320 mV, 10 mA cm^−2^) [19], NiCo_2_S_4_ nanoflakes (319 mV, 100 mA cm^−2^) [16], and Au@CoFeO_x_ (328 mV, 10 mA cm^−2^) [9]. The Tafel slopes of Au@NiCo_2_S_4_ NPs and NiCo_2_S_4_ NPs are shown in Figure 5b. Compared with bare NiCo_2_S_4_ NPs (49.1 mV dec^−1^), the Tafel slope of Au@NiCo_2_S_4_ (44.5 mV dec^−1^) is lower, indicating that Au@NiCo_2_S_4_ NPs possess a superior reaction kinetic, and a faster mass/electron transport rate. The Tafel slopes of these two samples are comparable or superior to other OER catalysts such as NiCo_2_S_4_ nanoflakes (53.3 mV dec^−1^) [16], NiCo_2_S_4_ (54.9 mV dec^−1^) [6], Ni_3_Se_2_ (40.2 mV dec^−1^) [13], NiCo_2_S_4_@NiFe LDH (46.3 mV dec^−1^) [23] and P-Co-Ni-S nanosheets (61.1 mV dec^−1^) [42]. All these results demonstrate that, due to the interaction between Au and NiCo_2_S_4_, Au@NiCo_2_S_4_ NPs exhibit better OER catalytic activity than bare NiCo_2_S_4_ NPs.

To further analyze the electronical factors affecting the OER activity of the samples, the EIS of Au@NiCo_2_S_4_ and bare NiCo_2_S_4_ NPs were measured. The diameter of the Nyquist plots of Au@NiCo_2_S_4_ is smaller than that of bare NiCo_2_S_4_, as shown in Figure 5c. The equivalent circuit, fitted according to the EIS, is shown in the inset of Figure 5c. The charge transfer resistance *R_t_* corresponds to the diameter of the Nyquist plot, and represents the resistance of mass transfer during the OER catalytic reaction, which is the decisive parameter affecting OER catalytic efficiency [43]. It can be seen that the R*_t_* of Au@NiCo_2_S_4_ (1.6 Ω) is lower than that of bare NiCo_2_S_4_ (2.0 Ω). The decrease of *R_t_* is due to the interaction between the Au NP and the NiCo_2_S_4_ shell, which can enhance the conductivity and shorten the length of charge diffusion [39]. The double-layer capacitance (C*_d_*) of the NPs surface is reflected by the *Y*_0_ value of the CPE, which represents the location of the absorbed molecules, and is related to the electrochemical surface area [36]. The *Y*_0_ value of Au@NiCo_2_S_4_ NPs is much larger than that of bare NiCo_2_S_4_ NPs, as shown in Table S4, indicating that its effective surface area is larger than that of bare NiCo_2_S_4_ NPs, and that Au@NiCo_2_S_4_ core-shell NPs have more active sites and exhibit better OER activity. In addition, as demonstrated by the XPS results, in Au@NiCo_2_S_4_ NPs, the proportion of high-valance Ni and Co cations is increased. The high-valance Ni and Co cations are the active sites for OER, so the Au@NiCo_2_S_4_ core-shell NPs possess better OER catalytic activity than bare NiCo_2_S_4_ [6]. Therefore, benefiting from the lower R*_t_*, larger effective surface area, the increased proportion of high-valence Ni and Co cations, and the single crystal NiCo_2_S_4_ shells, the Au@NiCo_2_S_4_ NPs possess better OER activity than bare NiCo_2_S_4_. As shown in Figure 5d, to test the stability of the materials, a current-time curve for the as-prepared NPs was characterized. Au@NiCo_2_S_4_ and bare NiCo_2_S_4_ have relatively excellent OER catalytic stability, maintaining 98.9%, and 95.2% after 5 h, respectively. This indicates that Au@NiCo_2_S_4_ and bare NiCo_2_S_4_ NPs have a superior stability over a long period of OER catalysis. The stability of Au@NiCo_2_S_4_ core-shell NPs is superior to NoCo_2_S_4_ NPs, which should be attributed to the following factors: (1) The introduction of Au cores reduced the overpotential, thus reducing the driving force for the reconstruction of NiCo_2_S_4_ [9]. (2) The interface interaction between Au and NiCo_2_S_4_ increased the proportion of Co^3+^ and Ni^3+^, thus enhancing the charge density at the specific domains and increasing the number of active sites [44]. (3) The larger effective surface area of Au@NiCo_2_S_4_ makes it easier for bubbles to detach from the electrode [45].

## 4. Conclusions

In summary, Au@NiCo_2_S_4_ and bare NiCo_2_S_4_ NPs were controllably prepared. The Au@NiCo_2_S_4_ sample exhibited a core-shell heterostructure with single crystal shells. The OER catalytic results confirm that the core-shell structure exhibits superior OER catalytic performance to bare NiCo_2_S_4_ NPs, which may be due to the interaction between Au and NiCo_2_S_4_. The XPS and EIS results demonstrated that the electronic conductivity, and the ratio of high-valance Ni and Co, were improved with the Au-NiCo_2_S_4_ interaction, which are beneficial to its electrocatalytic performance. The overpotential and Tafel slope of the Au@NiCo_2_S_4_ heterostructure NPs is 299 mV at 10 mA cm^−^^2^ and 44.5 mV dec^−^^1^, respectively. The construction of metal-semiconductor core-shell heterostructure will provide more chances for its application in the field of electrochemical water splitting.

## Supplementary Materials

The following are available online at https://www.mdpi.com/2079-4991/10/4/611/s1, Figure S1. TEM images of Au@NiCo_2_S_4_ NPs evolution experiment. Table S1. The ratios of elements at different times of Au@NiCo_2_S_4_ NPs evolution experiment. Figure S2. EDS spectra of the Au@NiCo_2_S_4_ and bare NiCo_2_S_4_ NPs. Table S2. Atomic ratio of Au, Ni, Co and S of Au@NiCo_2_S_4_ and bare NiCo_2_S_4_ NPs calculated by EDS. Figure S3. Angle-dependent TEM characterization of Au@NiCo_2_S_4_ NPs verifying the core-shell of the products. The shells produced in the outer shell of the NP were reasoned from the carbon deposit induced by the long-time high-energy electron irradiation. Scale bar 20 nm. Figure S4. HAADF-STEM images and STEM-EDS mappings for the elements Au, Ni, Co, and S of Au@NiCo_2_S_4_ NPs. Figure S5. XPS survey spectra of Au@NiCo_2_S_4_ and bare NiCo_2_S_4_ NPs. Figure S6. High resolution XPS spectra for the (a) S 2p, (b) Au 4f of Au@NiCo_2_S_4_ and bare NiCo_2_S_4_ NPs. Table S3. XPS peaks area ratio of Ni, and Co of Au@NiCo_2_S_4_ and bare NiCo_2_S_4_ NPs. Table S4. Electrochemical and equivalent circuit simulation parameters of the samples including Au@NiCo_2_S_4_, and bare NiCo_2_S_4_ NPs.
