# Supplementary material for: Enhanced OER Performances of Au@NiCo2S4 Core-Shell Heterostructure
Source: Nanomaterials (Basel). 2020 Mar 27;10(4):611. doi: 10.3390/nano10040611 (PMC7221621; doi:10.3390/nano10040611)
Supplement: Supplementary file 1 [file nanomaterials-10-00611-s001.pdf]

Supplementary Materials

Enhanced OER Performances of $Au@NiCo_2S_4$ Core-Shell Heterostructure

Yuepeng Lv †, Sibin Duan †, Yuchen Zhu, Peng Yin, and Rongming Wang *

Beijing Advanced Innovation Center for Materials Genome Engineering, Beijing Key Laboratory for Magneto-Photoelectrical Composite and Interface Science, School of Mathematics and Physics, University of Science and Technology Beijing, Beijing 100083, China; b20150331@xs.ustb.edu.cn (Y.L.); sibinduan@ustb.edu.cn (S.D.); cy931122@live.com (Y.Z.); yinpeng0537@163.com (P.Y.)

* Correspondence: rmwang@ustb.edu.cn

† These authors contributed equally to this work.

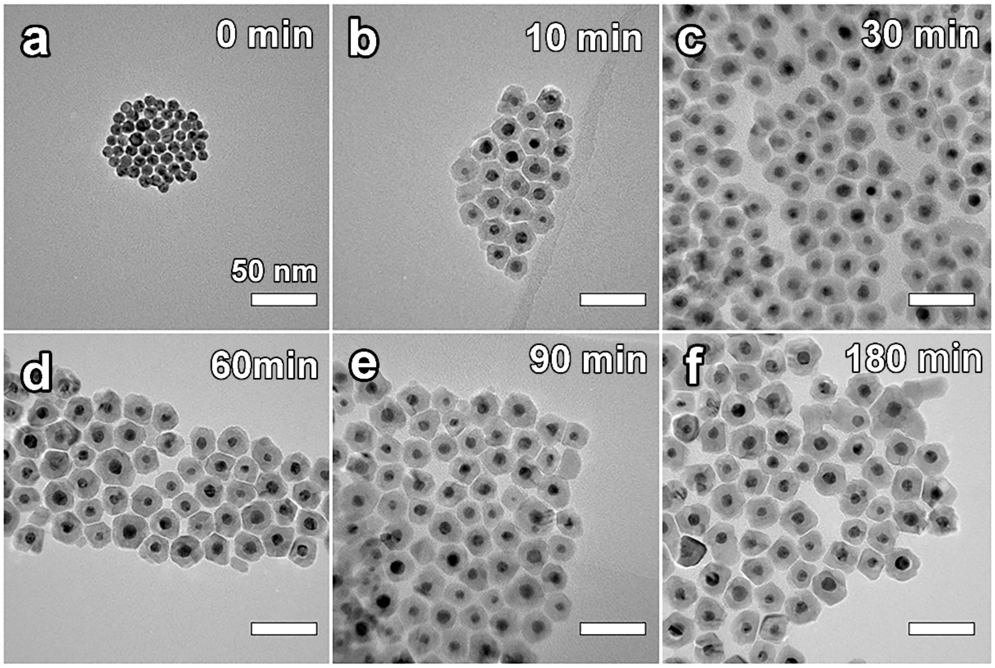

Figure S1. TEM images of Au@$NiCo_2S_4$ NPs evolution experiment.

Table S1. The ratios of elements at different times of Au@$NiCo_2S_4$ NPs evolution experiment.

Time (min)	Au (%)	Ni (%)	Co (%)	S (%)	Au:Co	Co:S
0	100	0	0	0	1:0	-
10	6.9	12	26.8	54.3	0.26:1	0.49:1
30	6.4	11.6	24.2	57.8	0.26:1	0.42:1
60	5.8	11.2	23.8	59.2	0.24:1	0.40:1
90	5.9	10.8	24.2	59.1	0.24:1	0.41:1
180	5.3	10.1	24.7	59.9	0.21:1	0.41:1

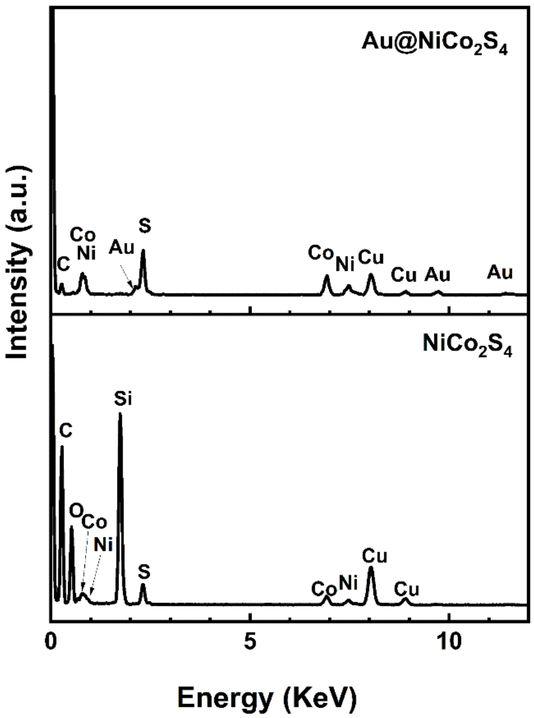

Figure S2. EDS spectra of the Au@NiCo$_2$S$_4$ and bare NiCo$_2$S$_4$ NPs.

Table S2. Atomic ratio of Au, Ni, Co and S of Au@$NiCo_2S_4$ and bare $NiCo_2S_4$ NPs calculated by EDS.

Sample	Au (%)	Ni (%)	Co (%)	S (%)
Au@$NiCo_2S_4$	5.3	10.1	24.7	59.9
$NiCo_2S_4$	0	11.4	25.5	63.1

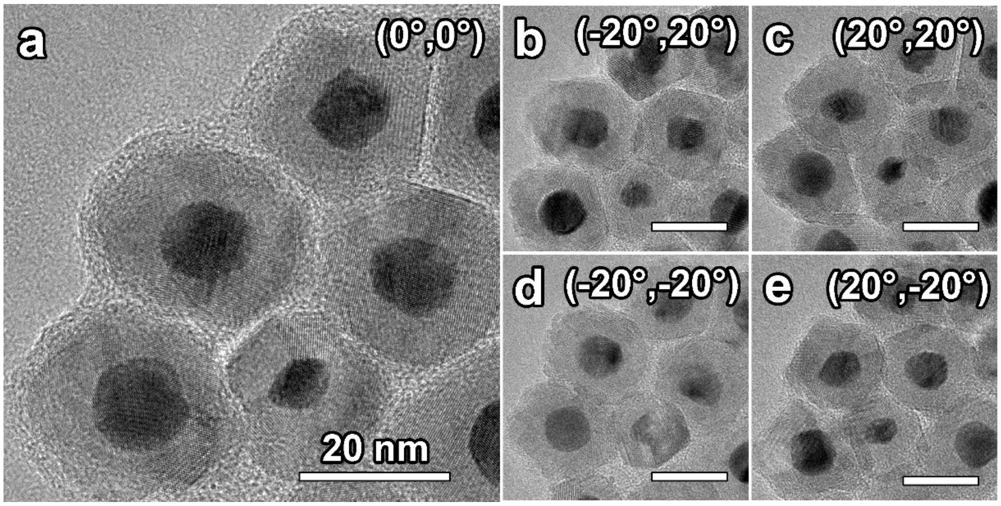

Figure S3. Angle-dependent TEM characterization of Au@$NiCo_2S_4$ NPs verifying the core-shell of the products. The shells produced in the outer shell of the NP were reasoned from the carbon deposit induced by the long-time high-energy electron irradiation. Scale bar 20 nm.

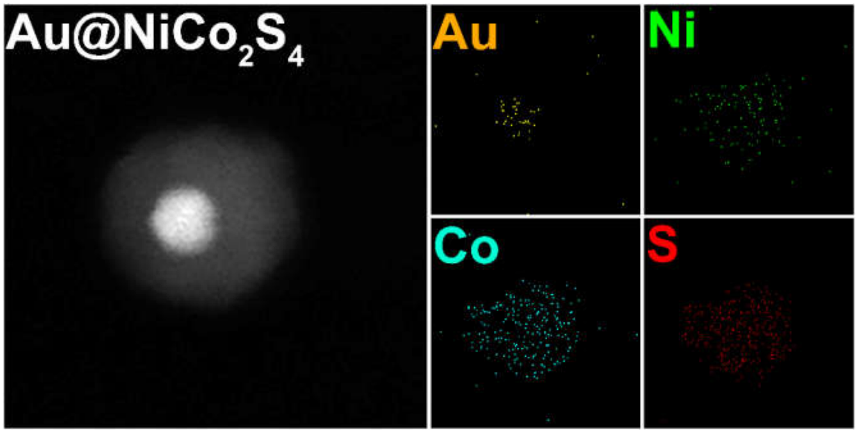

Figure S4. HAADF-STEM images and STEM-EDS mappings for the elements Au, Ni, Co, and S of Au@$NiCo_2S_4$ NPs.

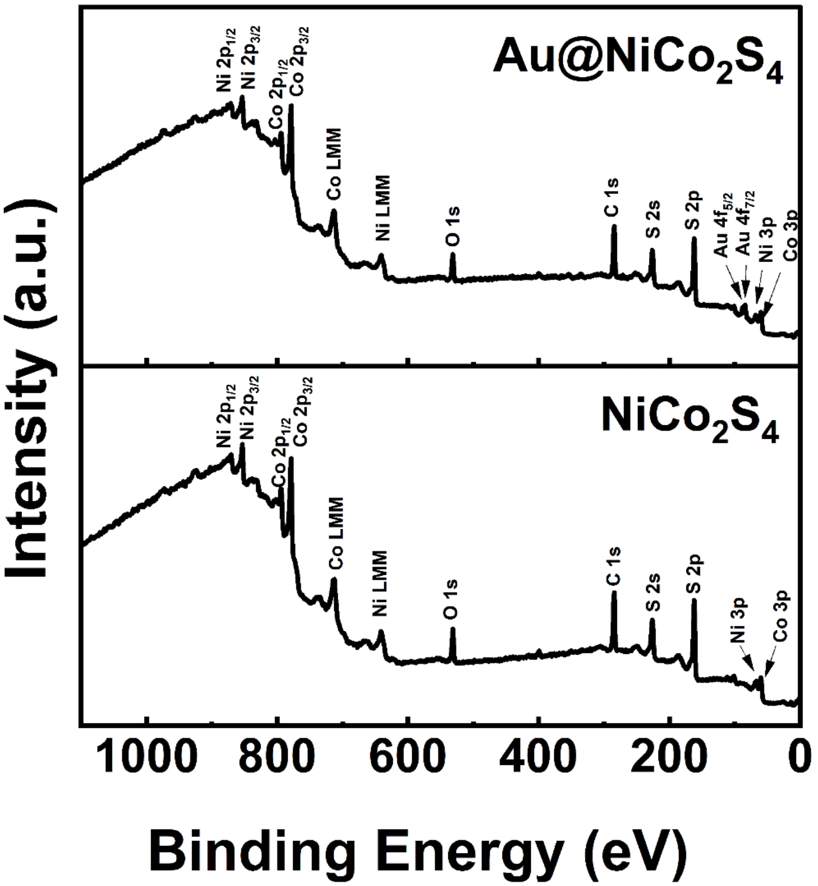

Figure S5. XPS survey spectra of Au@$NiCo_2S_4$ and bare $NiCo_2S_4$ NPs.

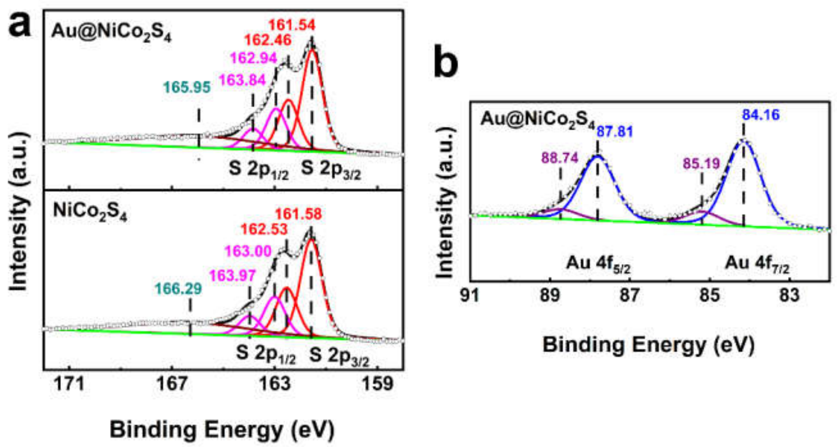

Figure S6. High resolution XPS spectra for the (a) S 2p, (b) Au 4f of Au@$NiCo_2S_4$ and $NiCo_2S_4$ NPs.

Table S3. XPS peaks area ratio of Ni, and Co of Au@$NiCo_2S_4$ and bare $NiCo_2S_4$ NPs.

Sample	Au@$NiCo_2S_4$	$NiCo_2S_4$
Ni^{2+}	47.5 %	51.4 %
Ni^{3+}	52.5 %	48.6 %
Co^{2+}	48.7 %	50.8 %
Co^{3+}	51.3 %	49.2 %

Table S4. Electrochemical and equivalent circuit simulation parameters of the samples including Au@$NiCo_2S_4$ and bare $NiCo_2S_4$ NPs.

**Sample**	**Au@$NiCo_2S_4$**	**$NiCo_2S_4$**
Overpotential at j = 10 $mA \cdot cm^{-2}$ (V)	0.299	0.312
Tafel slope ($mV \cdot dec^{-1}$)	44.5	49.1
Electrolyte resistance R_s (Ω)	2.7	2.7
Charge-transfer resistance R_t (Ω)	1.6	2.0
CPE:Y_0 (S·sn)	0.080	0.058
CPE:n	0.68	0.62
